# Inhibition of Rho GTPases in Invertebrate Growth Cones Induces a Switch in Responsiveness to Retinoic Acid

**DOI:** 10.3390/biom9090460

**Published:** 2019-09-07

**Authors:** Alysha Johnson, Tamara I. N. Nasser, Gaynor E. Spencer

**Affiliations:** Department of Biological Sciences, Brock University, 1812 Sir Isaac Brock Way, St Catharines, ON L2S 3A1, Canada

**Keywords:** axon guidance, Vitamin A, cytoskeleton, retinoid

## Abstract

During development, growth cones are essential for axon pathfinding by sensing numerous guidance cues in their environment. Retinoic acid, the metabolite of vitamin A, is important for neurite outgrowth during vertebrate development, but may also play a role in axon guidance, though little is known of the cellular mechanisms involved. Our previous studies showed that retinoid-induced growth cone turning of invertebrate motorneurons requires local protein synthesis and calcium influx. However, the signalling pathways that link calcium influx to cytoskeletal dynamics involved in retinoid-mediated growth cone turning are not currently known. The Rho GTPases, Cdc42 and Rac, are known regulators of the growth cone cytoskeleton. Here, we demonstrated that inhibition of Cdc42 or Rac not only prevented growth cone turning toward retinoic acid but could also induce a switch in growth cone responsiveness to chemorepulsion or growth cone collapse. However, the effects of Cdc42 or Rac inhibition on growth cone responsiveness differed, depending on whether the turning was induced by the all-*trans* or 9-*cis* retinoid isomer. The effects also differed depending on whether the growth cones maintained communication with the cell body. These data strongly suggest that Cdc42 and Rac are downstream effectors of retinoic acid during growth cone guidance.

## 1. Introduction

Retinoic acid has been implicated in the development [1,2] and regeneration [3,4] of various organ systems in several species. This includes the nervous system [5], where it can exert trophic effects to initiate and maintain neurite outgrowth [6,7,8,9]. Many effects of retinoic acid occur as a result of binding to nuclear receptors, the retinoic acid receptors (RARs) and retinoid X receptors (RXRs), which then regulate gene transcription. However, in addition to its transcriptional activity, retinoic acid can also exert non-genomic actions, either through retinoid receptors localized in non-nuclear cellular domains [10], or by binding directly to intracellular signalling molecules [11,12]. Examples of fast, non-genomic retinoid actions include the regulation of homeostatic plasticity in the hippocampus [10,13], modulation of cell firing and intracellular calcium [14,15] as well as chemotropic effects inducing growth cone turning of regenerating neurites [16].

The ability of retinoic acid to exert chemotropic effects was first demonstrated in developing neurites of chick embryonic neural tube cells [17], and later in regenerating newt spinal cord explants [8], where neurites grew toward the source of retinoic acid (chemoattraction). However, it was using regenerating cultured motorneurons from the mollusc *Lymnaea stagnalis* that it was determined that the chemoattractant effects of retinoic acid were non-genomic in nature [16]. The growth cones of regenerating molluscan neurons can be physically transected from the cell bodies and continue to grow for many hours. Importantly, these isolated growth cones retain their chemoattractive response to retinoic acid. Consistent with the findings of many other locally acting guidance molecules, the growth cone turning mediated by retinoic acid also requires local protein synthesis [16]. However, the identities of locally synthesized proteins are not yet known. Growth cone calcium levels are often an important determinant in growth cone responses to various guidance cues, and the same appears to be true for retinoic acid. In the presence of the calcium channel blocker cadmium, growth cone turning toward retinoic acid is significantly reduced or abolished [16]. However, the downstream signalling cascades which might link calcium influx to regulation of the cytoskeleton are not currently known, but potential candidates include Rho GTPases.

Rho GTPases are well known to mediate growth cone responses to various guidance cues, including netrin [18] and brain-derived neurotrophic factor (BDNF) [19]. They are a family of small guanosine triphosphate (GTP)-binding proteins that include cell division control protein 42 (Cdc42), Ras-related C3 (Rac) and Ras homolog (Rho). These binding proteins act as molecular switches to control signal transduction in the growth cone by cycling between a GDP-bound inactive form and a GTP-bound active form. Their activity is also tightly regulated by guanine nucleotide exchange factors (GEFs), GTPase-activating proteins (GAPs) and guanine nucleotide dissociation inhibitors (GDIs). Whereas Rho activation is often involved in repulsive turning responses or growth cone collapse [20], activation of Cdc42 and Rac is often required during chemoattractive growth cone responses. For example, Rac mediates growth cone attraction to netrin in rat embryonic spinal cord explants [18] and perturbing Cdc42 activity in cultured *Xenopus* spinal neurons abolishes chemoattraction induced by BDNF [19].

These Rho GTPases can be temporally and/or spatially regulated within the growth cone and can thus contribute to signalling pathways that link changes in growth cone calcium levels to the regulation of cytoskeletal dynamics required for directional changes [21]. As such, we hypothesize that they will play an important role in mediating the chemoattractive effects of retinoic acid. The aim of this study was to pharmacologically inhibit the Rho GTPases, Cdc42 and Rac, in order to determine their role in the chemoattractive growth cone responses of regenerating *Lymnaea* motorneurons to applied retinoids. We examined both biologically active retinoid isomers, all-*trans* retinoic acid (atRA) and 9-*cis* retinoic acid (9-*cis* RA), as both exist in the *Lymnaea* CNS, but very little is known of the role of 9-*cis* RA in neurite outgrowth or pathfinding. We performed the growth cone turning assays on both intact regenerating neurites, as well as growth cones isolated from the cell body (in order to determine any localized effects). We provide evidence that Cdc42 and Rac inhibition not only inhibited chemoattraction, but also induced a chemorepulsive response. However, the effects of Cdc42 or Rac inhibition differed, depending on the retinoid isomer applied, as well as whether the growth cone maintained communication with the cell body.

## 2. Materials and Methods

### 2.1. Animals

*Lymnaea stagnalis* were reared and housed in open air tanks containing aerated filtered water. Water was supplemented with Instant Ocean Sea Salt at a concentration of 0.6 g/L. Animal nutrition consisted of romaine lettuce, Spirulina fish food and carrot shavings. All animals used for cell culture experiments ranged from 16 to 20 mm in length.

### 2.2. Cell Culture Procedures

Animals were anaesthetized (25% Listerine^®^ in saline) and their CNS removed. The CNS were passed through a series of three, 10 min antibiotic saline (ABS) washes. Next, the CNS were treated with trypsin (Sigma-Aldrich; 6 mg in 3 mL Defined Medium (DM; comprised of 50% Leibowitz’s L-15 media and additional salts)) for 19.5 to 22 min, followed by a trypsin inhibitor (Sigma-Aldrich; 6 mg in 3 mL DM) for 10 min. The CNS were then pinned out in high osmolarity DM (800 μL of 1 M glucose in 30 mL DM) and the outer connective tissue and inner sheath surrounding the left and right Pedal ganglia were removed.

Individual Pedal A (PeA) motorneurons were chosen for these studies as they have been shown to generate extensive neurite outgrowth in cell culture [16,22], and have been used in previous growth cone studies examining the effects of retinoic acid in *Lymnaea* [23,24]. The PeA cells were removed from the left and right Pedal ganglia using a fire-polished glass pipette coated with Sigmacote (Sigma-Aldrich) to prevent cell adhesion. Suction was applied using a micrometer syringe to remove individual cell bodies from the ganglia and these cells were then plated into poly-l-lysine coated culture dishes containing 2.5 mL of Conditioned Medium (CM) and 0.5 mL of DM. *Lymnaea* CM contains unidentified trophic factors that are required for generating outgrowth in vitro [25]. AtRA was added to the culture dishes at the end of cell plating (10^−7^ M; final bath concentration) to promote neurite outgrowth. Extensive outgrowth was observed within 16 to 18 h following plating.

### 2.3. Chemicals

AtRA and 9-*cis* RA were obtained from Sigma-Aldrich and were prepared in ethanol (EtOH) and diluted using DM to a concentration of 10^−5^ M (0.1% final EtOH concentration) in the pipette. Vehicle control experiments used a concentration of 0.1% EtOH in the pipette. A selective, small-molecule inhibitor of Cdc42, ML141 (Sigma-Aldrich), (also known as CID2950007) [26] was prepared in dimethyl sulfoxide (DMSO; occasionally with Tween-80) and diluted using DM to produce a final bath concentration of 10^−5^ M [27] (0.1% final DMSO concentration). It has previously been shown (using a fibroblast cell line) that the inhibitory effects of 10^−5^ M ML141 were selective toward Cdc42 (with no inhibition of Rac) [26]. The vehicle was prepared in the exact same manner, in the absence of ML141. NSC23766 (Sigma-Aldrich), a selective, small-molecule inhibitor of Rac [28], was prepared in sterile distilled water and diluted using DM to produce a final bath concentration of 10^−4^ M [29]. 

### 2.4. Growth Cone Turning Assays

All growth cone assays were conducted in a similar manner as previously described [16,23,24]. Only active growth cones exhibiting a steady growth trajectory were used for growth cone turning assays. A retinoic acid isomer (atRA or 9-*cis* RA), or vehicle (EtOH), was pressure-applied (3–10 hPa) to one side of the growth cone via a pipette (up to ~6 μm in diameter), using an Eppendorf–Femtojet. The pipette was placed between 50 and 175 μm from the growth cone (depending on the size of the pipette), and the pressure applied was adjusted accordingly. A holding pressure of 1–2 hPa was used to prevent backflow into the pipette between applications. To examine the role of the Rho GTPases, Cdc42 and Rac, in retinoic acid-mediated growth cone turning, a pharmacological inhibitor of either Cdc42 (ML141) or Rac (NSC23766) was bath applied at least 1 h prior to growth cone turning assays. A gradient of retinoid (atRA or 9-*cis* RA) or vehicle, was then applied to growth cones that continued to actively grow in the presence of the inhibitor.

In order to examine the role of Cdc42 and Rac in isolated growth cone responses, the neurites were transected from the cell body using a sharp glass electrode. The isolated growth cones were monitored and only those with sustained activity fifteen min after transection were used for growth cone turning assays. A gradient of retinoic acid (or vehicle) was applied in a similar manner as described previously for intact growth cones. The transected neurites were monitored throughout the entire experiment to ensure no contact was re-established with the cell body (or any adjacent neurites attached to the cell body).

It should be noted that variability in individual growth cone responses to application of the retinoid and vehicle (seen previously and in this study) are common and likely due to differences in susceptibility to pressure application (artifact), differences in the size and motility of individual growth cones, as well as slight variations in experimental conditions across experiments (size and location of pipette). Additional factors which might have affected isolated growth cone responses include the extent of membrane, cytoplasm and/or organelles remaining in the isolated neurite to contribute to growth.

### 2.5. Growth Cone Measurements

All images were captured using a Zeiss Axiovert 200 inverted microscope and Retiga Exi camera with QCapture Suite 2.90.1 software (Quantitative Imaging Corporation). Individual growth cone turning angles were determined by measuring the angle between the growth cone’s initial trajectory and the final trajectory of that growth cone following application of a retinoid (or vehicle). These angles were determined by an individual blind to the condition of the experiment. An attractive turning response consisted of the growth cone turning toward the pipette and is indicated by a positive angle. Growth cone turning away from the pipette is indicated by a negative angle.

When examining the effect of the Rho GTPase, Rac, in response to atRA, the length of neurite extension was also measured using Northern Eclipse imaging software (Empix imaging, ON). Growth cone advancement was measured as the distance travelled by the tip of the growth cone at the beginning of the assay to its final location at the end of the experiment (a period of 50 to 60 min). An increase in the length of the neurite by the end of the experiment produced a positive value whereas neurite retraction produced a negative value.

### 2.6. Data and Statistical Analysis

Statistical analyses were preformed using SigmaStat software and graphs were generated using Graphpad Prism, Version 8.0 for Mac OS X. A one-way analysis of variance (ANOVA) was performed on growth cone data sets, followed by a Tukey-Kramer *post hoc* test. Data were expressed as mean ± standard error of mean (SEM) and were deemed significant when *p* < 0.05.

## 3. Results

### 3.1. The Inhibition of Cdc42 Induces a Switch in Growth Cone Responsiveness to AtRA, but not to 9-cis RA

It has previously been shown that calcium influx is required for retinoid-induced growth cone turning [16]. As Rho GTPases are known downstream effectors of calcium and are involved in growth cone turning responses to other guidance cues [30,31], we first investigated the role of the Rho GTPase, Cdc42, in retinoid-mediated growth cone turning.

**Figure 1:**
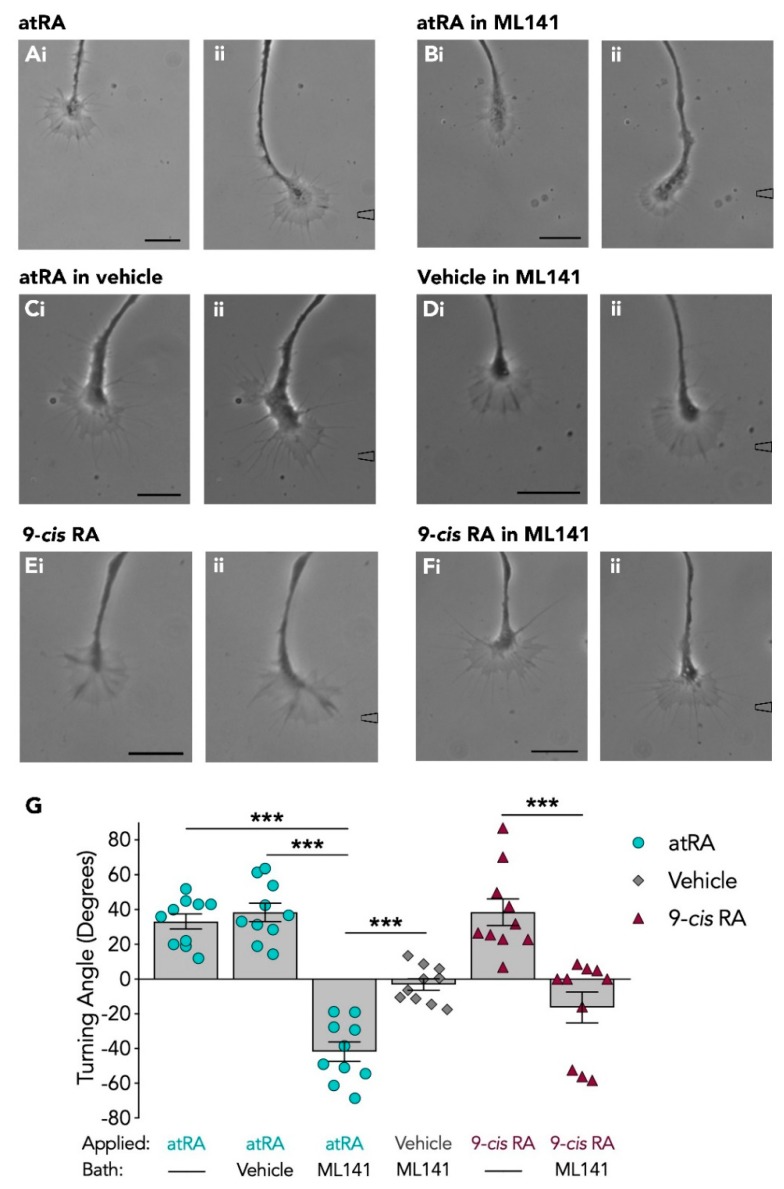
Inhibiting Cdc42 switches the atRA, but not 9-*cis* RA-induced growth cone response from attraction to repulsion. The first image of each data set (**A**–**Fi**) illustrates the growth cone prior to application of the retinoid/vehicle (t = 0), whereas the second image of each data set (**A**–**Fii**) represents the growth cone response. The approximate location of the pipette containing either retinoid or vehicle is shown to the right of an image (**A**–**Fii**) and times (t) of growth cone responses are given in minutes. Application of atRA induced attractive growth cone turning in normal conditions (**Ai**–**ii**; *n* = 10; t = 53), as well as in the presence of the vehicle in the bath (**Ci**–**ii**; *n* = 10; t = 24). This attractive growth cone turn was abolished and converted to a repulsive turn (away from pipette) in the presence of the Cdc42 inhibitor, ML141 (**Bi**–**ii**; *n* = 10; t = 50). Vehicle (applied via pipette) in the presence of ML141 did not induce significant turning responses (**Di**–**ii**; *n* = 10; t = 31). Application of 9-*cis* RA also induced attractive growth cone turning (**Ei**–**ii**; *n* = 10; t = 12) which was blocked in the presence of ML141 (**Fi**–**ii**; *n* = 10; t = 21). Scale bars: 15 µM. (**G**) Graph depicting the mean turning angles of growth cones in response to retinoid (or vehicle) in the presence or absence of the Cdc42 inhibitor, ML141 (*** *p* < 0.001).

A local gradient of atRA (10^−5^ M in pipette) was first applied to the growth cones of regenerating motorneurons. Focal application of atRA produced attractive growth cone turning of regenerating neurites (mean turning angle: +33.2° ± 4.3; *n* = 10). A representative example of a PeA growth cone responding to atRA is shown in Figure 1A. In contrast, application of the vehicle alone (control) failed to induce growth cone turning toward the pipette, producing a mean turning angle of −5.2° ± 4.5 (*n* = 8; data not shown), consistent with previous findings [9,32]. In the presence of the Cdc42 inhibitor, ML141 (10^−5^ M; final bath concentration), the growth cones failed to turn toward the source of atRA (mean turning angle: −41.7° ± 5.6; *n* = 10) and a representative example is shown in Figure 1B. The Cdc42 inhibitor used in these studies was dissolved in DMSO (0.1%; final bath concentration), but in the presence of the vehicle alone, growth cones continued to turn toward the focally applied atRA (mean turning angle: +38.5° ± 5.3; *n* = 10; Figure 1C).

Interestingly, the inhibition of Cdc42 not only prevented growth cone turning toward atRA, but also appeared to convert the atRA-induced attractive response to a repulsive response. A switch in response from attraction to repulsion may enable growth cones to respond differently to the same guidance molecule at different stages during nervous system development. It is however possible that this switch in responsiveness was not cue-specific, but rather that the Cdc42 inhibitor increased growth cone susceptibility to the pressure of chemical application from the pipette. Further control experiments were thus performed in which only the vehicle (EtOH) was applied to growth cones in the presence of the Cdc42 inhibitor, ML141. Growth cones failed to turn toward the source of the vehicle (as expected), and only a slightly negative turning angle was observed in response to the vehicle in the presence of the Cdc42 inhibitor (mean turning angle: −3.1° ± 3.3; *n* = 10; Figure 1D). The mean turning angles in each condition are summarized in Figure 1G. A one-way ANOVA revealed a significant effect (*F*_(5,54)_ = 30.119, *p* < 0.001), and a Tukey Kramer *post-hoc* test determined there was a significant reduction in positive growth cone turning toward atRA in the presence of the Cdc42 inhibitor, compared to in its absence (*p* < 0.001). The negative turning angle elicited by atRA in ML141 was also significantly different from that produced by application of EtOH (vehicle) in the presence of ML141 (*p* < 0.001), indicating that the switch in responsiveness to repulsive growth cone turning was likely a specific response to atRA.

We next tested the growth cone responsiveness to the isomer 9-*cis* RA, as very little is currently known about the role of 9-*cis* RA in either neuronal regeneration or growth cone guidance. Both retinoid isomers were previously shown to exert similar chemoattractive effects on *Lymnaea* growth cones [9,32], though isomer-dependent effects can also occur [14,15]. As expected, 9-*cis* RA elicited growth cone attraction (mean turning angle: +38.5° ± 7.7; *n* = 10; Figure 1E), which was similar to that produced by atRA (Figure 1A,G). In the presence of the Cdc42 inhibitor, ML141, this attractive turning response was completely abolished (mean turning angle: −16.3° ± 8.8; *n* = 10; Figure 1F). However, this negative turning angle produced by 9-*cis* RA in ML141 was not significantly greater than that produced by application of the vehicle (EtOH) alone in the presence of ML141 (*p* = 0.654), and so we cannot conclude that 9-*cis* RA induced a switch in responsiveness following Cdc42 inhibition.

Overall, these data showed that inhibition of Cdc42 abolished growth cone attraction to the retinoid isomers. Inhibition of Cdc42 also induced a switch in growth cone responsiveness to atRA (but not 9-*cis* RA), from an attractive turning response to a repulsive growth cone turning response. 

### 3.2. The Cdc42 Inhibitor Blocks Retinoic Acid-Induced Growth Cone Turning of Isolated Growth Cones

An advantage of using cultured *Lymnaea* neurons is that neurites can survive transection and growth cones isolated from the cell body continue to grow for many hours. We have previously shown that retinoic acid-induced growth cone turning is transcriptionally independent and relies on local protein synthesis. That is, growth cones physically isolated from the cell body continue to show attractive responses to retinoic acid, which are blocked in the presence of a protein synthesis inhibitor [16]. Accordingly, our next aim was to use isolated growth cones to examine the involvement of Cdc42 in localized signalling mechanisms within the growth cone that are independent of communication with the cell body.

**Figure 2:**
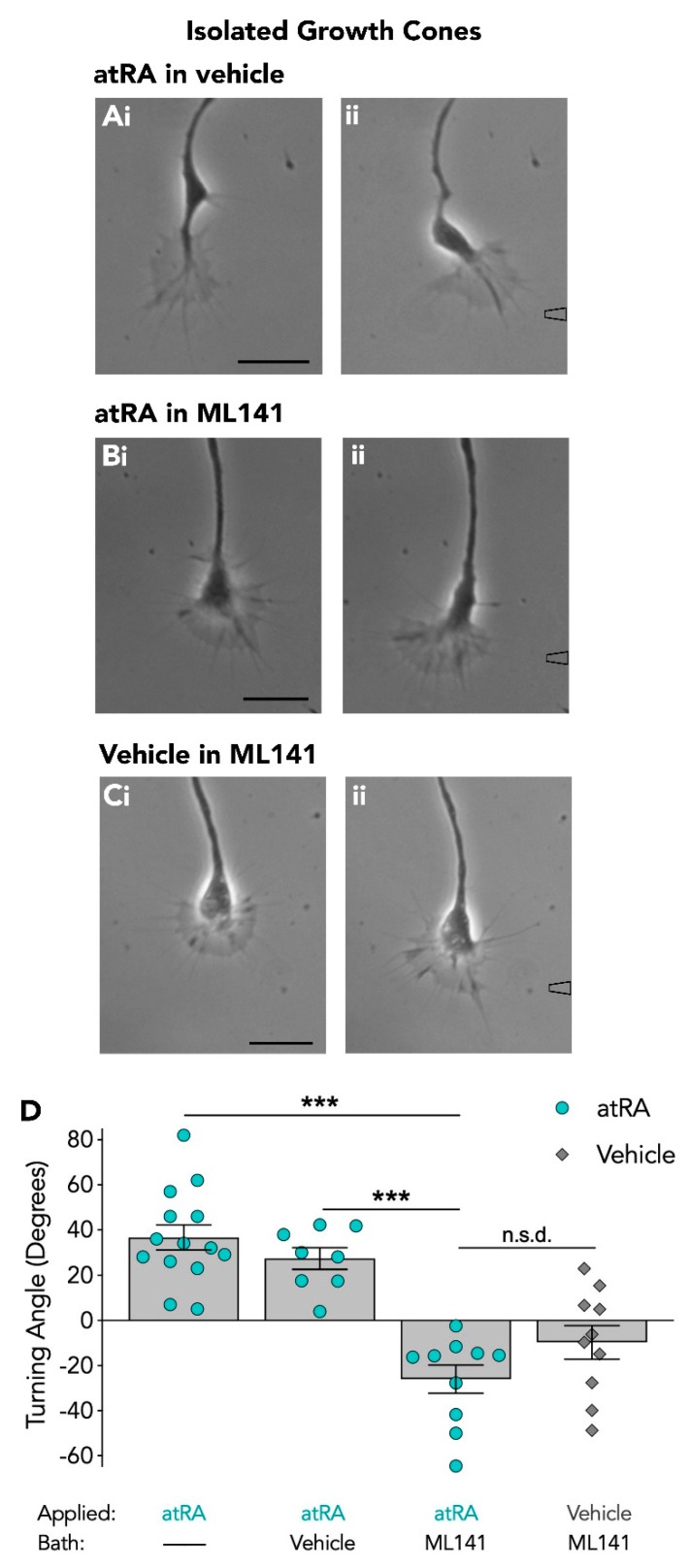
Isolated growth cones fail to turn toward atRA in the presence of the Cdc42 inhibitor. Representative images depicting the turning response of isolated growth cones to atRA in the presence of the vehicle (**Ai**–**ii**; *n* = 8; t = 24), and the failure to turn toward atRA in the presence of the Cdc42 inhibitor, ML141 (**Bi**–**ii**; *n* = 10; t = 29). The isolated growth cone response to focal application of the vehicle alone in the presence of ML141 is also shown (**Ci**–**ii**; *n* = 10; t = 20). The approximate location of the pipette containing either atRA or vehicle is shown to the right of an image (**A**–**Cii**) and times (t) of growth cone responses are provided in minutes. Scale bars: 15 µM. (**D**) Graph depicting the mean turning angles of isolated growth cones in each condition. Data were expressed as the mean ± SEM and analyzed using a one-way ANOVA (*F*_(3,38)_ = 23.959, *p* < 0.001) followed by a Tukey Kramer *post-hoc* test. The mean turning angle of isolated growth cones to atRA is significantly different in the presence of ML141 compared to in the presence of the vehicle (*** *p* < 0.001). The mean turning angle produced by atRA in ML141 was not significantly different (n.s.d.) than the mean turning angle produced by the vehicle in ML141.

Growth cones were completely isolated from the cell body and given at least 15 min to recover from injury. The turning responses of isolated growth cones to atRA (10^−5^ M in pipette) were first tested and as expected, isolated growth cones responded and turned toward atRA (+36.6° ± 5.6; *n* = 14). In the presence of the vehicle (DMSO), isolated growth cones also continued to turn toward the focally applied atRA with a mean turning angle of +27.3° ± 4.8 (*n* = 8; Figure 2A). However, in the presence of the Cdc42 inhibitor, isolated growth cones failed to initiate a turn toward the focally applied atRA (mean turning angle: −26.0° ± 6.3; *n* = 10; Figure 2B). In order to determine whether this was again, a switch in responsiveness of the growth cone, we applied the vehicle (EtOH) alone to the growth cone in the presence of ML141, and growth cones failed to turn toward the vehicle as expected, producing a mean turning angle of −9.8° ± 7.4 (*n* = 10; Figure 2C). However, there was no significant difference between the turning angle to atRA and to the vehicle in ML141 (*p* = 0.286; Figure 2D). Thus, even though it was clear that inhibition of Cdc42 by ML141 blocked attractive growth cone turning induced by atRA, it was more difficult to determine whether a switch in responsiveness occurred in isolated growth cones following inhibition of Cdc42. However, it should be noted that despite the smaller negative turning angle of isolated growth cones (compared to intact growth cones) in ML141, 8 of 8 isolated growth cones turned *toward* atRA in the absence of ML141, whereas 10 of 10 turned *away* from atRA in the presence of ML141. Overall, these data indicate a requirement for Cdc42 in retinoid-mediated growth cone attraction of both intact and isolated growth cones.

### 3.3. The Rac Inhibitor Induced a Switch in Growth Cone Responsiveness to AtRA, but not to 9-cis RA

**Figure 3:**
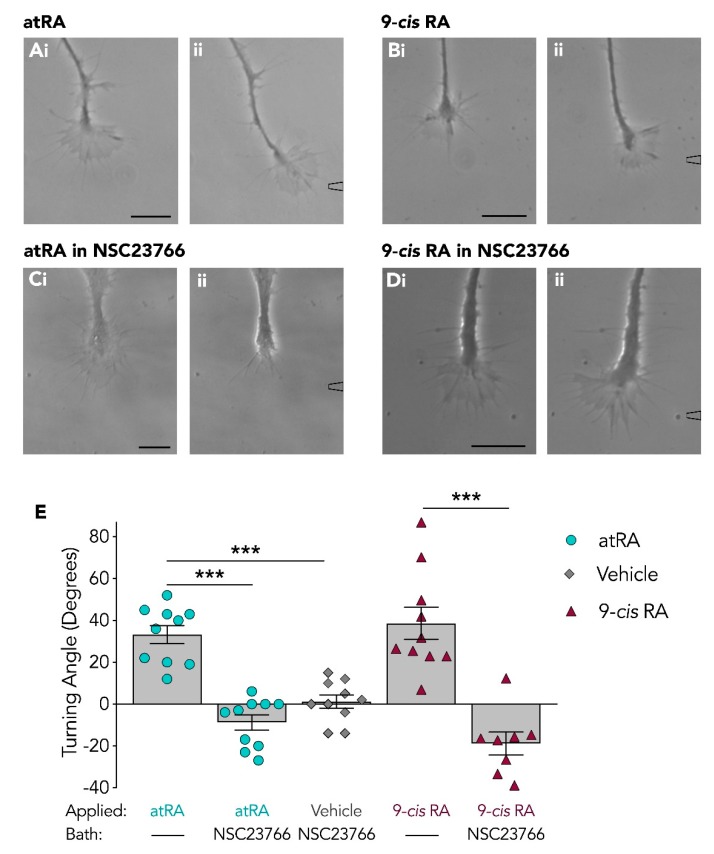
Growth cone turning mediated by retinoids is blocked by the inhibition of Rac. Application of atRA (**Ai**–**ii**; *n* = 10; t = 23) and 9-*cis* RA (**Bi**–**ii**; *n* = 10; t = 19) induced attractive growth cone turning. In the presence of the Rac inhibitor, NSC23766, growth cones failed to turn toward either atRA (**Ci**–**ii**; *n* = 10; t = 30) or 9-*cis* RA (**Di**–**ii**; *n* = 8; t = 14). Scale bars: 15 µM. (**E**) Graph depicting the mean turning angle of growth cones in response to application of atRA or 9-*cis* RA, both in the presence and absence of NSC23766. The turning response to the vehicle in NSC23766 is also given. NSC23766 significantly reduced the mean turning angle of growth cones in response to each retinoid (*** *p* < 0.001). NSC23766 did not however induce significant differences in growth cone turning responses to retinoid application compared to vehicle application.

We next sought to investigate the requirement for Rac in retinoid-induced growth cone turning. As previously indicated (in Figure 1), a local gradient of either atRA or 9-*cis* RA produced attractive growth cone turning with a mean turning angle of +33.2° ± 4.3 (*n* = 10) or +38.5° ± 7.7 (*n* = 10), respectively (examples shown in Figure 3A,B). The growth cone turning responses toward atRA or 9-*cis* RA were next examined in the presence of the Rac inhibitor, NSC23766 (10^−4^ M; final bath concentration). In contrast to the Cdc42 inhibitor used in this study, the Rac inhibitor was dissolved in sterile water, thus additional bath control (vehicle) experiments were not required.

In the presence of the Rac inhibitor (NSC23766), growth cones failed to turn toward atRA exhibiting a mean turning angle of −8.8° ± 3.7 (*n* = 10). The inhibition of growth cone turning toward atRA is clearly depicted by the representative images shown in Figure 3C. Moreover, in the presence of the Rac inhibitor, growth cones also failed to turn toward 9-*cis* RA, producing a mean turning angle of −18.8° ± 5.5 (*n* = 8; Figure 3D). A summary of the mean growth cone responses to atRA and 9-*cis* RA in the presence and absence of NSC23766 are shown in Figure 3E. Statistical analysis was performed using a one-way ANOVA and revealed a significant effect (*F*_(4,43)_ = 24.334, *p* < 0.001). A Tukey Kramer *post-hoc* test showed that there was a significant reduction in growth cone turning toward atRA in the presence of the Rac inhibitor, compared to in its absence (*p* < 0.001). Furthermore, the growth cone turning angle in response to 9-*cis* RA was significantly different in the presence of the Rac inhibitor as compared to its absence (*p* < 0.001). Overall, these findings indicate that Rac may have an important role in mediating growth cone turning toward retinoids.

**Figure 4:**
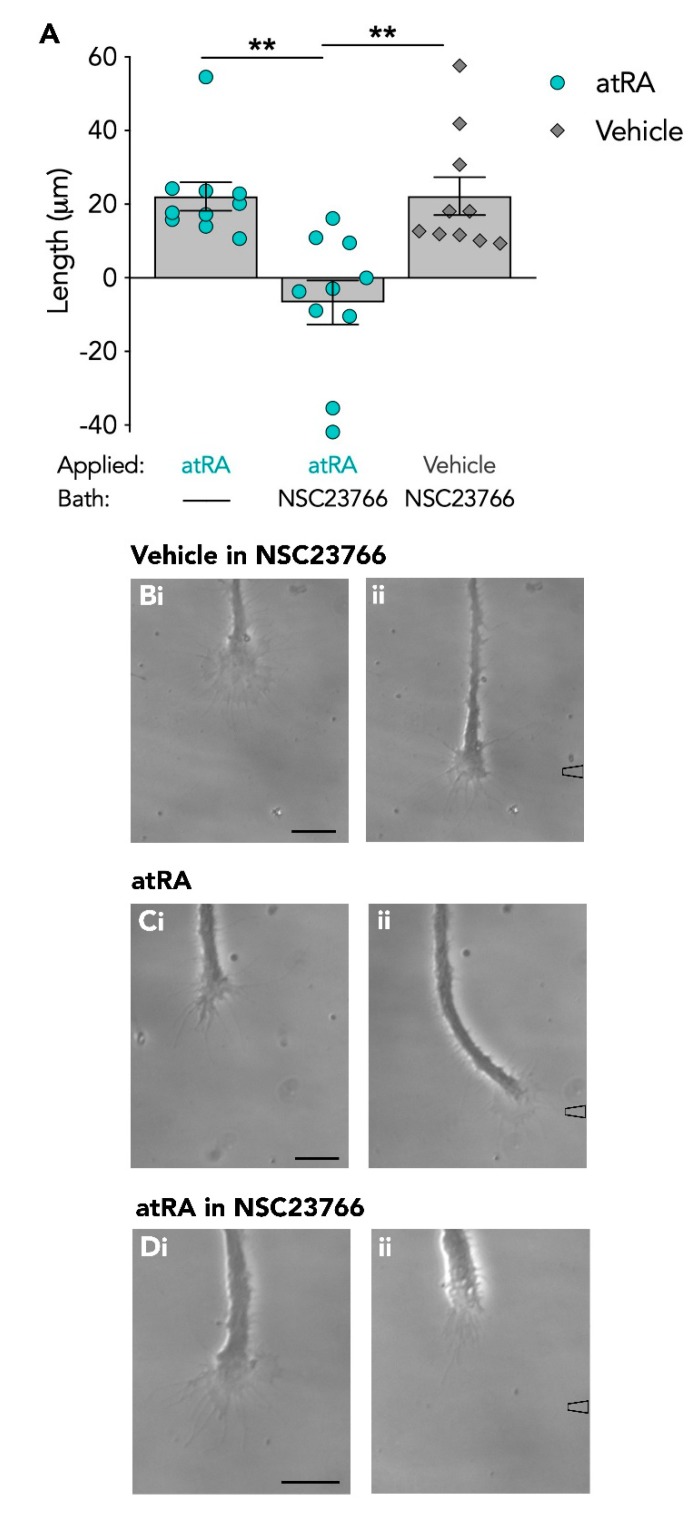
Inhibiting Rac switches the atRA-induced growth cone attraction to collapse and retraction. (**A**) Graph depicting the length of growth cone advancement in response to atRA (or vehicle) in the presence or absence of the Rac inhibitor, NSC23766. In the presence of the Rac inhibitor, growth cone advancement following application of atRA was inhibited, compared to all other experimental conditions (** *p* < 0.01). In response to application of vehicle in the presence of NSC23766, growth cones did not turn, but continued to advance (**Bi**–**ii**; *n* = 10; t = 40). (**C**–**D**) Example of an individual growth cone that first produced attractive growth cone turning to atRA (**Ci**–**ii**; t = 60), but following application of NSC23766 to the bath, later collapsed and retracted in response to atRA (**Di**–**ii**; t = 55). Scale bars: 15 µM.

In addition to preventing the chemoattractive response to atRA, the Rac inhibitor also appeared to induce a switch in the growth cone responsiveness to atRA, though in this instance, not by causing a significant repulsive turning angle, but by inducing growth cone collapse and in particular, neurite retraction. Growth cones showed a collapsed morphology and neurites retracted during the application of atRA, a behaviour indicative of an avoidance response to a chemorepulsive guidance cue. When we determined the extent of movement of growth cones in the presence (Figure 3C) or absence (Figure 3A) of the Rac inhibitor, NSC23766, we found that growth cones exposed to atRA in NSC23766, showed an overall mean retraction of −6.7 ± 6.0 μm (*n* = 10), whereas in the absence of NSC23766, growth cones continued to advance over a mean distance of +22.1 ± 3.9 μm (*n* = 10) over the same time period (Figure 4A). It is also possible, however, that the presence of the Rac inhibitor may have sensitized the growth cones to the pressure of chemical application and that the growth cone collapse observed was not a switch in responsiveness to atRA. In order to rule out this possibility, control experiments were performed, in which the vehicle (EtOH) alone was applied to the advancing growth cones in the presence of NSC23766. Though the growth cones did not exhibit any turning response to the vehicle, as expected (mean turning angle: +1.2 ± 3.1°; *n* = 10), the growth cones continued to actively advance over a distance of +22.2 ± 5.1 μm (*n* = 10; Figure 4B) and did not demonstrate any growth cone collapse or retraction following application of the vehicle solution alone. These data suggest that the growth cone collapse in response to atRA in the presence of NSC23766 was not merely due to increased sensitivity to pressure (Figure 4B). A summary comparing the distance of growth cone advancement in response to atRA, in the presence or absence of the Rac inhibitor, (as well as in response to the vehicle) is shown in Figure 4A. A one-way ANOVA revealed a significant effect (*F*_(2,27)_ = 10.751; *p* < 0.001) and a Tukey Kramer *post-hoc* test confirmed a significant reduction in growth cone advancement in response to atRA in the presence of the Rac inhibitor, compared to in its absence (*p* = 0.001).

To further confirm that the growth cone collapse was induced only in the presence of the Rac inhibitor, we determined the response to atRA of the *same* growth cone, first in the absence and then presence of the Rac inhibitor. Figure 4C illustrates the typical positive growth cone turning response and growth advancement initially obtained in response to atRA (in the absence of the inhibitor) followed by the pronounced growth cone collapse and retraction that occurred in response to atRA in the presence of the Rac inhibitor (Figure 4D). These data strongly suggest that the Rac inhibitor did indeed cause a switch in growth cone response from attraction to growth cone collapse and neurite retraction in response to atRA. However, this same neurite retraction was not observed in any growth cones in response to 9-*cis* RA. It should be noted that even though 7 of 8 growth cones turned away from 9-*cis* RA following Rac inhibition (as opposed to 10 of 10 turning toward 9-*cis* RA with no Rac inhibitor), the mean repulsive turning angle was not significantly different from that produced following application of the vehicle alone in the Rac inhibitor (Figure 3E). As such, we cannot conclude that 9-*cis* RA induced a switch in responsiveness to mediate repulsion, despite most growth cones showing some turning away from the retinoid source.

In summary, these data indicate that Rac is required for attractive growth cone turning toward both retinoid isomers, as this was abolished in the presence of the Rac inhibitor. The Rac inhibitor also induced a switch in growth cone responsiveness to atRA (but not 9-*cis* RA), from chemoattraction to growth cone retraction.

### 3.4. Isolated Growth Cones Continue to Turn Toward Retinoids Following Rac Inhibition

**Figure 5:**
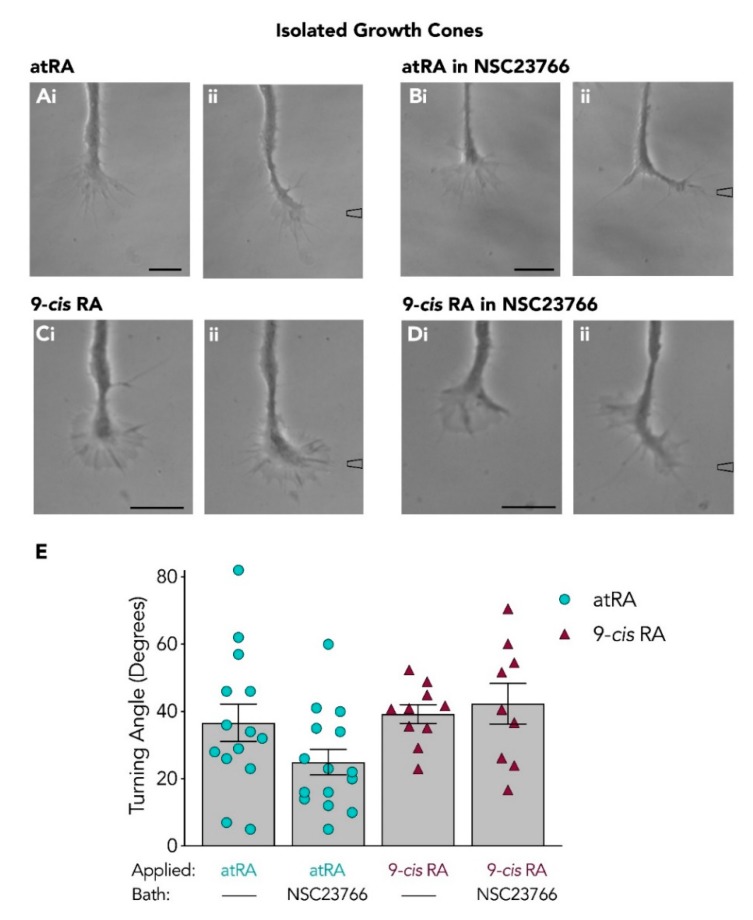
Isolated growth cones continue to turn toward retinoids in the presence of the Rac inhibitor. Representative images depicting the turning response of isolated growth cones to atRA (**Ai**–**ii**; *n* = 14; t = 30) or 9-*cis* RA (**Ci**–**ii**; *n* = 10; t = 18) in the absence of the Rac inhibitor, NSC23766. In the presence of NSC23766, isolated growth cones continued to turn toward both atRA (**Bi**–**ii**; *n* = 15; t = 40) and 9-*cis* RA (**Di**–**ii**; *n* = 9; t = 15). Scale bars: 15 µM. (**E**) Graph depicting the mean turning angles of isolated growth cones in response to atRA or 9-*cis* RA, in the presence or absence of NSC23766.

Similar to our studies with the Cdc42 inhibitor, we next examined whether the isolated growth cone responses to atRA (Figure 5A) or 9-*cis* RA (Figure 5C) would also be abolished following Rac inhibition. The responses of isolated growth cones to atRA (10^−5^ M in pipette) were thus tested again in the presence of the Rac inhibitor, NSC23766, and interestingly, these isolated growth cones continued to turn and advance toward the focally applied atRA (Figure 5B), with a mean turning angle of +24.9° ± 3.8 (*n* = 15). Because this isolated growth cone response to atRA was very different from that obtained from the intact neurites, we also next determined whether the isolated growth cone turning changed in response to the other retinoid isomer, 9-*cis* RA. Indeed, the isolated growth cones also continued to turn toward 9-*cis* RA in the presence of the Rac inhibitor (+42.3° ± 6.1; *n* = 9; Figure 5D). These turning angles are summarized in Figure 5E and a one-way ANOVA revealed no significant differences in the turning angles of isolated growth cones, either in the absence or presence of NSC23766 (*F*_(3,44)_ = 2.709; *p* = 0.057).

Taken together, these studies have shown that Rac inhibition induces a switch in growth cone response (of intact growth cones) to atRA from chemoattraction to retraction, but did not induce the same effects on growth cone responses to the 9-*cis* RA isomer. Moreover, the Rac inhibitor did not significantly inhibit growth cone turning of isolated growth cones toward either atRA or 9-*cis* RA. These data suggest that the involvement of Rac in growth cone turning differs depending on whether the turning is induced by the atRA or 9-*cis* RA isomer, but also, more importantly, depends on whether the growth cones have maintained communication with the cell body.

Overall, we have provided evidence for a role of Rho GTPases in retinoid-induced growth cone guidance. Evidence has been provided that the inhibition of Cdc42 or Rac prevents growth cone turning toward retinoic acid. Interestingly, the inhibition of Cdc42 and Rac not only blocked growth cone turning toward atRA, but also induced a switch in growth cone responsiveness from chemoattraction to chemorepulsion in intact growth cones. Moreover, the inhibition of Rac did not inhibit growth cone turning of isolated growth cones, suggesting its involvement depends on whether the growth cones have maintained communication with the cell body. These results therefore strongly suggest that the signal transduction pathways underlying retinoic acid-mediated growth cone guidance involve the Rho GTPases, Cdc42 and Rac.

## 4. Discussion

Retinoic acid is a molecule that has been implicated in several developmental and regenerative processes, including its actions as a chemoattractant to guide neurites in vertebrates [8,17] and invertebrates such as *Lymnaea stagnalis* [9,32]. Similar to many other guidance cues, we have previously shown that retinoid-induced growth cone attraction requires both local protein synthesis and calcium influx [16]. However, the signalling cascades which might link calcium influx to the regulation of cytoskeletal dynamics involved in retinoid-mediated chemoattraction have not previously been studied. Rho GTPases are known downstream effectors of calcium and have emerged as important regulators of the actin cytoskeleton. Here, we now provide evidence for the role of the Rho GTPases, Cdc42 and Rac, in retinoid-mediated growth cone attraction.

Cdc42 and Rac have been previously associated with promoting neurite outgrowth and inducing positive growth cone turning responses [33,34] and their disruption can lead to axon pathfinding defects [34]. For example, disruption of Rac was found to result in growth cone pathfinding defects of *Drosophila* embryonic motor axons [30]. Interestingly, expression of either constitutively active or dominant-negative forms of Rho GTPases both cause guidance errors, indicating that very precise regulation of Rho GTPases is crucial for appropriate growth cone behaviour and navigation [35,36,37].

In this study, we showed that inhibiting both Cdc42 and Rac blocked growth cone turning toward a gradient of retinoic acid, suggesting a role for both Rho GTPases in this chemoattractive response. The attractive guidance cue BDNF similarly activates both Cdc42 and Rac [19], and it has been proposed that these GTPases share common up or downstream effectors. Importantly, the inhibition of Cdc42 and Rac in this study not only blocked retinoic acid-induced growth cone attraction, but induced a switch in responsiveness from chemoattraction to chemorepulsion, at least for atRA. Similarly, findings in *C. elegans* indicated that lowering levels of the Rac homolog, ced-10, led to a switch in Semaphorin-1 responsiveness of migrating cells from attraction to repulsion [38].

Numerous studies indicate that whether a cue is attractive or repulsive can be modulated by various factors and that a switch in responsiveness may allow growth cones to respond differently to the same guidance molecule at different stages of development or regeneration. Studies in *Xenopus* growth cones have shown that inhibition of cyclic adenosine monophosphate (cAMP) or cAMP-dependent kinases (such as PKA), causes a switch from attraction to repulsion in response to either netrin [39,40] or BDNF [19,41]. As there is evidence that Rac activation can be controlled by cAMP/PKA signalling [42], it is possible that the switch in response to atRA observed here may also result from an interaction between Rac and cAMP levels within the growth cone. It is thought that cAMP facilitates bidirectional growth cone responses by the modulation of calcium channel activity, which in turn, allows for differential activation of calcium-dependent effector proteins [40,43]. It is likely that numerous calcium-dependent effector proteins act upstream of Rho GTPases and these may include calcium-calmodulin-dependent protein kinase II (CaMKII) and/or protein kinase C (PKC). Signal transduction from calcium to Cdc42 in netrin-induced growth cone guidance in *Xenopus* spinal neurons is mediated by PKC and requires basal activity of CaMKII [21]. It is therefore conceivable that CaMKII and PKC may act upstream of Cdc42 to control the direction of growth cone turning induced by retinoic acid.

Notably, following inhibition of Cdc42 or Rac, only the atRA isomer (not 9-*cis* RA) induced a switch in growth cone responsiveness to produce repulsive turning or growth cone retraction. AtRA and 9-*cis* RA are both biologically active isomers of retinoic acid which have been detected in the *Lymnaea* CNS at relatively similar concentrations [32]. However, despite both inducing similar chemoattractive effects in *Lymnaea* neurons, they can also exert different effects on the firing properties of *Lymnaea* motorneurons [14]. Furthermore, atRA significantly reduces the voltage-gated calcium current [44] and intracellular calcium levels of cultured *Lymnaea* somata [15], whereas 9-*cis* RA does not. As the microdomain concentration and localization of calcium is an important determinant of growth cone responses to many guidance cues [43,45], it is possible that the switch in growth cone responsiveness to atRA during Cdc42 and Rac inhibition, is a result of changes in growth cone calcium signalling and/or differential activation of calcium-dependent proteins in response to atRA, but not to 9-*cis* RA (or at least, not to the same extent). Finally, it should be noted that not only do the Rho GTPases function downstream of calcium to mediate growth cone turning, but can themselves also influence calcium dynamics [21].

An interesting finding from this study was that either the involvement of Rac, or the actions of the inhibitor, differed depending on whether the growth cones maintained their communication with the cell body. That is, retinoid-induced chemoattraction of intact growth cones was blocked by Rac inhibition, but the isolated growth cones continued to respond to both retinoid isomers in the presence of the Rac inhibitor. GEFs promote the conversion of GDP to GTP to enhance Rac activation [33]. The Rac inhibitor used in our experiments, NSC23766, binds to Rac and has been shown to competitively inhibit the interaction between Rac and a subset of its GEFs, Tiam1 and Trio, thus preventing their interaction and preventing activation of Rac. However, there are other GEFs that have been shown to activate Rac [46] and one of these (Vav) is described as a promiscuous GEF that can bind to Rac using an alternative mechanism [28]. It is thus possible that alternative GEFs, perhaps upregulated or activated following neurite transection, allowed the activity of Rac to persist. As NSC23766 is a competitive inhibitor of Rac, it can be displaced and its inhibitory actions reduced when the levels of GEFs are increased [28]. Though we consider this unlikely, it is possible that GEF levels may have increased sufficiently to displace NSC23766 in the transected neurites.

It is also plausible that neurite transection might have impeded the effects of the Rac inhibitor by preventing the transport of certain signalling ligands or regulatory proteins (such as GEFs). The known abundance and diversity of the many GEFs suggests a complex regulatory network [33]. Indeed, it has been shown in bipolar *C. elegans* neurons that various GEFs can antagonize the effects of others in different neurite processes [47]. This might suggest that disruption of communication with the cell body, or even with other neurites, may have disrupted the regulatory interactions between various GEFs in the transected *Lymnaea* neurites.

Another plausible explanation is that neurite transection may have triggered a compensatory mechanism, either to activate Rac, or even different proteins/signalling pathways not normally involved in retinoic acid-mediated chemoattraction. In *Xenopus* neurons, BDNF appears to activate an alternative pathway which is less dependent on Cdc42 under certain pharmacological inhibitory conditions [19]. This alternate pathway appeared only when the activity of the more dominant Cdc42, as well as RhoA pathways were blocked, indicating that the balance of Rho GTPase activity could potentially unmask different signalling pathways. Furthermore, it has been shown that when the downstream target of Rac1, actin related protein 2/3 complex (Arp2/3) was partially inhibited resulting in growth cone collapse, feedback activation of Rac1 occurred, allowing growth cone recovery, even during persistent inhibition of Arp2/3 [48]. This suggests that feedback regulatory mechanisms likely exist in these signalling pathways that, under certain conditions (such as pharmacological inhibition), may become active, possibly reactivating the pathway (or even alternate pathways). Though it is well known that there is crosstalk between the individual Rho GTPases, extensive crosstalk between the regulatory proteins (as mentioned above for GEFs), has also been proposed. It is thus feasible that neurite transection triggers activation of different signalling pathways during Rac inhibition (than when neurites are intact), allowing the retinoid-induced chemoattraction to persist. As neurite transection also induces a large influx of calcium [49], it is also possible that activation of alternate signalling pathways results from this injury-induced spike in calcium levels. In summary, either reduced communication with the cell body, or an injury response from neurite transection, might have resulted in activation of a different or parallel mechanism to permit growth cone turning during Rac inhibition. Interestingly, when Cdc42 was inhibited, isolated growth cones failed to turn toward retinoic acid, perhaps suggesting the absolute requirement for Cdc42 in this retinoid-induced chemoattractive response. However, it should be noted that unlike the Rac inhibitor (NSC23766), the Cdc42 inhibitor, ML141, is an allosteric, non-competitive inhibitor [26] that displaces nucleotides from Cdc42. As such, if neurite transection increased GEF levels or induced alternate GEF activity, this would not have prevented the inhibitory action of ML141 in the same manner as it might have for NSC23766 [28]. Finally, it is not yet known what the downstream effectors of either Cdc42 or Rac are in this retinoid-mediated chemoattractive response, but potential effectors may include invertebrate homologues of WASP proteins (which promote actin formation [50]), or p21-acitvated kinases, whose substrates include cytoskeletal regulators [51].

The atRA and 9-cis RA-induced chemoattraction has previously been shown to be mimicked by many retinoid receptors agonists [23,52]. As this is also the case for transected neurites [23], it indicates a non-genomic role for retinoid receptors in growth cone turning. However, we cannot rule out the possibility that the chemoattractive effects of retinoic acid may not always involve the RXR and RAR. For example, there is evidence from other studies that retinoic acid directly modulates PKC, which possesses a retinoic acid-binding site [11,12]. There is also evidence that retinoic acid can activate the cAMP response element binding protein (CREB), independently of either RXR or RAR (at least in tracheobronchial epithelial cells) [53].

In *Lymnaea*, both the RXR and RAR have been cloned [23,24] and are present in the neurites and growth cones of these PeA motorneurons. It is proposed that attractive growth cone turning entails the accumulation of functional membrane receptors and cytoskeletal components on the side of the growth cone closest to the chemoattractant [54]. However, the retinoid receptors have been detected in both membrane and cytoplasmic compartments in *Lymnaea* neurons [23,24] and as retinoic acid can diffuse across neuronal membranes, the activation of receptors in this case, may occur internally in the growth cone, rather than in the membrane. It will thus be of interest to determine whether either of these retinoid receptors become asymmetrically distributed during attractive growth cone turning, and if so, whether the Rho GTPases are involved in facilitating their transport through cytoskeletal-dependent vesicle transport.

The process of growth cone guidance is one that is multifaceted, involving extracellular guidance cues, intracellular signalling cascades and cytoskeletal rearrangements. While progress has been made in the identification of numerous guidance molecules and their receptors, gaps still remain in the understanding of intracellular signalling pathways that regulate growth cone dynamics. Although much is known about the ability of retinoids to mediate neurite outgrowth during nervous system development, the cellular and molecular mechanisms underlying growth cone pathfinding by retinoids remain largely unknown. We have provided further evidence that many of the same signalling processes (such as local protein synthesis, calcium, and now Rho GTPases) utilized by traditional proteinaceous guidance cues, are also utilized by retinoic acid.

Overall, these studies have revealed a mechanism in which Cdc42 and Rac are downstream effectors of retinoic acid and regulate growth cone guidance. We demonstrated that the inhibition of Cdc42 and Rac not only blocked growth cone turning toward atRA and 9-*cis* RA, but in the case of atRA, also induced a switch in growth cone responsiveness from attraction to repulsion or collapse. Furthermore, we showed that the inhibition of Rac produced differing effects on intact or isolated growth cones, with isolated growth cones maintaining their response to retinoids. In conclusion, our findings indicate that the Rho GTPases, Cdc42 and Rac, control the directional motility of growth cones in response to retinoic acid.
